# Preliminary Report on the Utilization of Plastic Lymph as a Reparative Tissue in Abdominal Surgery

**Published:** 1904-03

**Authors:** E. C. Davis

**Affiliations:** Atlanta, Ga.; Gynecologist to Presbyterian Hospital, to Tabernacle Infirmary and Surgeon of the Halcyon Retreat and to McViccar Hospital for Colored Women


### PRELIMINARY REPORT ON THE UTILIZATION OF PLASTIC LYMPH AS A REPARATIVE TISSUE IN ABDOMINAL SURGERY.

By E. C. DAVIS, M.D., ATLANTA, GA.

Gynecologist to Presbyterian Hospital, to Tabernacle Infirmary and Surgeon of The Halcyon Retreat and to McViccar Hospital for Colored Women.

About a year ago while operating on a patient with an ectopic pregnancy about six months advanced, in which the fetal sac was firmly adherent to the abdominal viscera, an effort was made to detach a part of the sac wall that was attached to the small intestine. In this separation it was observed that the peritoneal coat of the intestine was being removed with the sac wall; this lead me to replace the sac wall together with the peritoneum and fasten it in place with fine silk sutures. The fetal side was touched with carbolic acid and alcohol. After removing the other portions of the gestation sac together with the fetus, the abdomen was closed without drainage. The recovery of this patient was almost uneventful and the tissue left must have been absorbed, as it gave no trouble.

Again several weeks ago, another case made it necessary to utilize similar tissue in the repair of an intestinal perforation, or rather as a splint to the tissue until nature could take care of the injuries beneath. The case was as follows: On opening the abdomen for an acute perforative appendicitis in which an abscess had formed and the cavity was filled with offensive pus and fecal contents, there being an opening in the intestine as large as the end of the index finger. Along the cecum was a dark grayish area where the appendix had lain, in an abnormal position, making the tissue appear soft, friable and unpromising for recovery. With considerable difficulty was the opening in the intestine closed, and the stitches placed in the healthy peritoneum appeared to be under much tension, so much so, that I feared lest they would cut through. Just over the area described was a thick piece of well-organized lymph which nature had thrown out to wall off the abscess and prevent it from invading the abdominal cavity. This was shaped so as to cover the area easily, and it occurred to me that by sterilizing the abscess side of this with carbolic acid and alcohol it could be used to brace this tissue and support the already strained stitches. This was done and it was turned down over the area and stitched with mattress sutures through the lymph and Lembert's through the peritoneum. The mattress sutures were used because the Lembert's cut through the friable lymph. This made an excellent approximation, but being a little in doubt about the security of this tissue it was deemed advisable to drain. The patient made an excellent recovery and I feel sure the abdomen could have been closed without drainage and the patient would have done equally well. There was no fecal fistula, the openings closed promptly, and his recovery was uninterrupted. I am unaware of any one having previously attempted to utilize this tissue for such purposes and would like to know if it has been done.

It strikes me that this principle can be utilized to shorten convalescence which would otherwise follow prolonged drainage.

My reasons for using this were as follows: (1) Nature suggests this in her efforts at repair, and in placing a barrier to check the extension of the infection. (2) The intestinal side of this tissue is usually sterile and that toward the abscess is readily made so after the manner already described. (3) It is abundantly present in these cases, fairly well nourished and easily adjusted. (4) It can be utilized as a splint to the tissues, until nature can make the necessary repairs to the tissues beneath, and then as a rule it is absorbed and put out of the way. (5) It prevents many of the annoying adhesions and places the tissues in a more nearly normal condition. (6) It may shorten a convalescence by the avoidance of drainage. (7) It prevents the occurrence of fecal fistulæ with their distressing sequellæ.

These are the chief reasons which now recommend this step to me, and I am not unmindful of the fact that my experience has not been sufficient to establish a rule, hence I give this to the profession to more carefully investigate before rendering a verdict.

